# A Single-Institution Cohort Study of Autologous Platelet-Rich Plasma Gel for Hard-to-Heal Chronic Wounds: Potential Role in Microvascular Regeneration

**DOI:** 10.3390/jcm15083120

**Published:** 2026-04-19

**Authors:** Miki Fujii, Kazuki Shimada, Takako Komiya, Hajime Matsumura

**Affiliations:** Department of Plastic and Reconstructive Surgery, Tokyo Medical University, Tokyo 160-0023, Japan

**Keywords:** chronic wounds, platelet-rich plasma, vasculitic ulcers, microcirculatory ischemia, angiogenesis, wound healing, chronic limb-threatening ischemia

## Abstract

**Background/Objectives:** Chronic wounds that fail to respond to standard wound care (SWC) remain a major clinical challenge. Platelet-rich plasma (PRP) is an advanced regenerative therapy that delivers platelet-derived growth factors involved in angiogenesis and tissue repair. However, clinical data in Asian populations and evidence regarding ulcers associated with vasculitis or microangiopathic ischemia remain limited. This study evaluated the efficacy, safety, and treatment frequency of autologous PRP gel prepared using the newly approved AutoloGel System^®^ in Japan. **Methods:** This single-center retrospective study included 20 patients with chronic ulcers unresponsive to ≥28 days of conventional therapy by a wound specialist. PRP gel was applied weekly for up to eight sessions under current insurance coverage. Primary outcomes were wound healing rate at 12 weeks after PRP initiation and healing duration. Healing time during specialist-directed conventional therapy was compared with that following PRP using the Wilcoxon signed-rank test. **Results:** Twenty patients (mean age 60 ± 15 years) with diverse refractory ulcers—including diabetic foot ulcers, chronic limb-threatening ischemia, vasculitic ulcers, venous leg ulcers, pressure ulcers, and surgical site infections—were analyzed. All wounds achieved complete epithelialization within 12 weeks. Healing time decreased significantly from 87.2 ± 77.1 days during conventional therapy to 47.9 ± 28.5 days after PRP initiation (median 60 vs. 44 days, *p* = 0.0107). No treatment-related adverse events were observed. **Conclusions:** Weekly autologous PRP gel therapy prepared using the AutoloGel System^®^ was associated with improved healing outcomes in refractory chronic wounds. Favorable outcomes were observed in traditionally difficult-to-treat conditions, including vasculitis-associated and microangiopathic ischemic ulcers. These findings suggest the potential role of PRP in promoting angiogenesis and improving microcirculatory perfusion in wounds associated with microvascular dysfunction.

## 1. Introduction

Chronic wounds represent a major global health problem associated with impaired physical function, reduced quality of life, and substantial healthcare costs. As populations age and the prevalence of diabetes and peripheral vascular disease increases, the incidence of chronic wounds continues to rise worldwide. Chronic wounds are characterized by a prolonged inflammatory phase and dysregulation of cytokines and growth factors involved in normal tissue repair, which prevents the wound from progressing through the physiological healing cascade [1]. Despite advances in wound management, including the widespread adoption of the TIME concept (tissue, infection/inflammation, moisture balance, and edge of the wound) for wound bed preparation, a significant proportion of wounds remain refractory to standard wound care [2,3]. In particular, tissue ischemia plays a critical role in delayed wound healing. Although macrovascular ischemia can often be corrected by revascularization procedures such as endovascular treatment (EVT) or bypass surgery, some wounds remain unhealed even after technically successful revascularization. This phenomenon is frequently attributed to persistent microcirculatory dysfunction, which limits tissue perfusion and angiogenesis at the wound site.

Platelet-rich plasma (PRP) has emerged as a regenerative therapy that delivers a concentrated mixture of platelet-derived growth factors and bioactive molecules involved in tissue repair [4,5,6,7,8,9]. Platelets release numerous growth factors stored in alpha granules, including vascular endothelial growth factor (VEGF), platelet-derived growth factor (PDGF), and transforming growth factor-β (TGF-β), which regulate angiogenesis, cell proliferation, and extracellular matrix formation. In addition to these growth factors, platelet degranulation also releases a variety of cytokines, chemokines, adhesion molecules, and antimicrobial proteins that contribute to inflammation modulation, cell migration, and host defense. Through these combined mechanisms, PRP may improve the wound microenvironment and promote tissue regeneration. Furthermore, accumulating evidence has highlighted the versatility of PRP across different conditions, including musculoskeletal injuries and experimental skin regeneration models, supporting its broad regenerative potential [8,9].

The efficacy of PRP therapy has been demonstrated in several types of chronic wounds, including diabetic foot ulcers, venous leg ulcers, and pressure ulcers [4,5,6,7]. However, clinical evidence remains limited for ulcers associated with vasculitis or microangiopathic ischemia. These conditions are characterized by inflammation or structural damage of the microvasculature, which impairs tissue perfusion and may limit the effectiveness of conventional treatments. Therefore, the present study aimed to evaluate the clinical outcomes of autologous PRP gel therapy prepared using the AutoloGel System^®^ in patients with refractory chronic wounds in an Asian population, with particular attention to wounds associated with vasculitis and microcirculatory ischemia.

## 2. Materials and Methods

### 2.1. Study Design

This was a single-center, retrospective study. From 16 January 2025 to 30 September 2025, a total of 29 patients (22 male, 7 female) with refractory ulcers treated with PRP gel at our institution were retrospectively reviewed. Among these, patients who met the predefined inclusion and exclusion criteria were included in the final analysis. PRP therapy was indicated for refractory ulcers that failed to respond to conventional therapy after at least 28 days of treatment. Conventional therapies were performed according to the TIME concept by a plastic surgeon specializing in wound healing. Among conventional therapies, basic fibroblast growth factor spray treatment (Fiblast^®^, Kaken Pharmaceutical Co., Ltd., Tokyo, Japan; bFGF) or negative pressure wound therapy (NPWT) for 4 weeks was provided when covered by insurance. Each specialist administered disease-specific treatments as follows: for venous leg ulcer (VLU), surgical treatment of varices, compression therapy, and leg elevation; for diabetic foot ulcer (DFU), glycemic control, infection management, and offloading with appropriate footwear; for ischemic foot ulcers, revascularization via EVT, or bypass surgery; and for vasculitic ulcers, autoimmune disease management and revascularization as appropriate. Failure of conventional therapy was defined as less than a 50% in wound size after ≥4 weeks of appropriate standard wound care (SWC).

The exclusion criteria were: (1) patients with known hypersensitivity to calcium chloride used in this product or to thrombin derived from bovine blood, or for whom these agents are contraindicated; (2) patients with severe hematologic disorders (e.g., leukemia, thrombocytopenia, coagulopathy, aplastic anemia, or severe anemia); (3) wounds associated with malignant tumors; (4) wounds with active infection; (5) progressive ischemia; (6) wound that cannot be appropriately offloaded; (7) patients unable to undergo weekly treatment; and (8) patients unable to stop smoking.

### 2.2. PRP Gel Preparation and Application

The AutoloGel System^®^ (Rohto Pharmaceutical Co., Ltd., Osaka, Japan) consists of a blood collection kit (blood collection tube and needle) and a medication set (2% calcium chloride, 5000 IU thrombin, and 250 mg/mL ascorbic acid), and requires a specialized centrifuge for preparation. The blood collection tubes contain citrate dextrose solution and solution A (ACD-A, sodium citrate hydrate, citrate hydrate, and glucose) as anticoagulants. Five milliliters of CaCl_2_ (Calcium chloride) was added to the thrombin and left for at least 5 min. For wounds smaller than 12.5 cm^2^, 5 mL of blood was collected from the patient, and for wounds between 12.5 and 25.0 cm^2^, 10 mL was collected. The collected blood was centrifuged at 4236× *g* for 30 s, and the PRP was separated from the whole blood. The PRP was then extracted, ascorbic acid was added, and thrombin and CaCl_2_ were mixed to activate the platelets and plasma, producing a PRP gel. PRP prepared with this system achieves a near-physiological platelet concentration (approximately 1.3 × baseline). Before applying the PRP gel, all slough or necrotic tissue in the wound was sharply debrided, with or without local anesthesia. The PRP gel was applied directly to the wound and covered with a dressing. Patients were instructed to remove the dressing and rinse the area at least 24 h post-application. PRP treatment was administered once weekly, with insurance coverage allowing up to two courses per patient (one course = 4 weeks). All procedures were performed in an outpatient setting equipped with a dedicated centrifuge, and SWC was provided between PRP sessions. The maximum number of PRP applications was set at eight (once weekly), based on the insurance coverage period. If the wound healed before the eighth application, PRP treatment was concluded.

### 2.3. Outcome Measures

**Figure 1:**
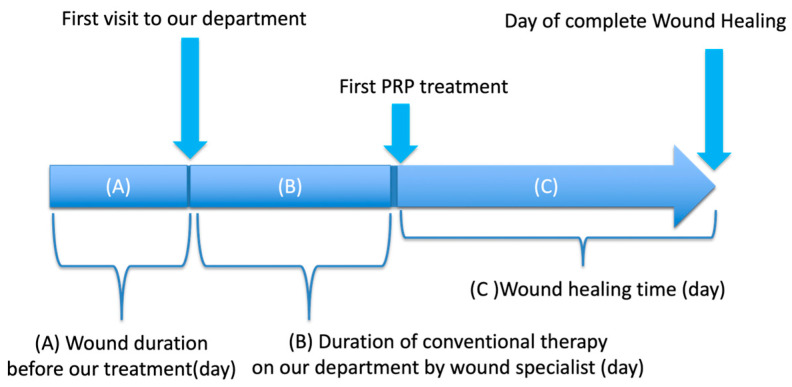
Assessment of each duration.

Primary outcomes were wound healing rate at 12 weeks after PRP initiation and healing duration. The wound duration before visiting our department (A), the duration of conventional therapy in our department by a wound specialist (B), the wound healing time following the initial PRP treatment (C) and the wound healing rate 12 weeks after the initial PRP treatment were assessed (Figure 1). Wound healing was defined as complete epithelialization of the wound by the corresponding author, and healing time was defined as the interval between the initial PRP application and complete epithelialization. The wound healing time (C) was also compared with the duration of conventional therapy administered by a wound specialist (B).

### 2.4. Statistical Analysis

Healing duration was compared between the conventional treatment period (B) and the period following the initiation of PRP therapy (C) in 20 cases of refractory ulcers. Because the sample size was relatively small and the distribution of healing time differences was not assumed to be normal, the Wilcoxon signed-rank test was used for the analysis. A *p*-value < 0.05 was considered statistically significant. This approach is consistent with previous studies recommending non-parametric methods for small sample sizes and non-normally distributed data [10].

### 2.5. Ethics Approval and Consent to Participate

This study was performed in accordance with the Declaration of Helsinki and was approved by the institutional ethics committee of Tokyo Medical University (Approval No. T2025-0133). As this retrospective study did not involve human biological specimens, the need for written informed consent was waived in line with the ethical guidelines for medical and health research involving human participants in Japan.

## 3. Results

### 3.1. Patient Characteristics

A total of 20 patients (15 male, 5 female; mean age 60 ± 15 years) were included in the final analysis (Table 1).

The ulcers were localized as follows: foot, 10; lower leg, 7; back, 1; chest, 1; and Ischium, 1. The diagnoses were diabetic foot ulcer (DFU), 4 (one patient also had vasculitis due to rheumatoid arthritis); chronic limb-threatening ischemia (CLTI), 3; venous leg ulcer (VLU), 3; SSI, 4; and pressure ulcer (PU), 1; vasculitic ulcer, 5; secondary to polyarteritis nodosa, 1; systemic lupus erythematosus, 1; schleroderma, 1; IgA nephropathy, 1; and ulcerative colitis, 1. Among the five patients with vasculitis, all were steroid users, and one was using immunosuppressants. None required revascularization, as the ischemia was due to vasculitis rather than peripheral arterial disease (PAD). Of the three patients with CLTI, one underwent revascularization with EVT, but the blood supply remained insufficient. The remaining patients with CLTI had no indications for revascularization due to microvascular disease. Before PRP treatment, all wounds had been managed by a wound specialist for 87.2 ± 77.1 days using conventional therapies, including bFGF, NPWT, and wound dressings, with a maximum ulcer size of 25 cm^2^ at initiation. Except for four patients (Cases 7, 9, 16 and 19), the other 16 were referred from another hospital or different departments within our university. Prior to referral, all patients had already undergone extended treatment periods, with 8 of 16 receiving more than 1 month of therapy, including three patients who had been treated for more than 1 year (Table 2).

### 3.2. Wound Outcomes (Table 2)

All wounds were healed within 12 weeks after the initial PRP treatment, with an average healing time of 47.9 ± 28.5 days. The mean number of PRP applications was 4.3. No adverse events directly related to PRP treatment were reported. No cases of severe infection occurred during the treatment period. In some patients, local signs of infection were observed and managed with oral antibiotics based on clinical findings and wound culture results. Appropriate offloading and activity restriction were implemented when indicated. In cases where infection was suspected, PRP therapy was temporarily discontinued and resumed after resolution of the infection.

**Figure 2:**
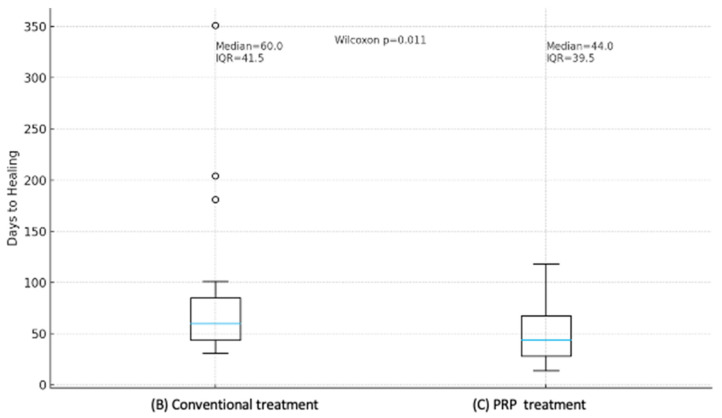
Comparison of healing duration between conventional treatment and PRP.

Because the distribution of paired differences was not assumed to be normal, the Wilcoxon signed-rank test was used. The median healing time decreased from 60.0 days (IQR 43.8–85.2) under SWC to 44.0 days (IQR 28.0–67.5) with PRP, demonstrating a statistically significant improvement (*p* = 0.0107) (Table 3 and Figure 2).

### 3.3. Cases

#### 3.3.1. Case 8: 65-Year-Old Female, Vasculitic Ulcer on the Right Lateral Malleolus (Figure 3)

**Figure 3:**
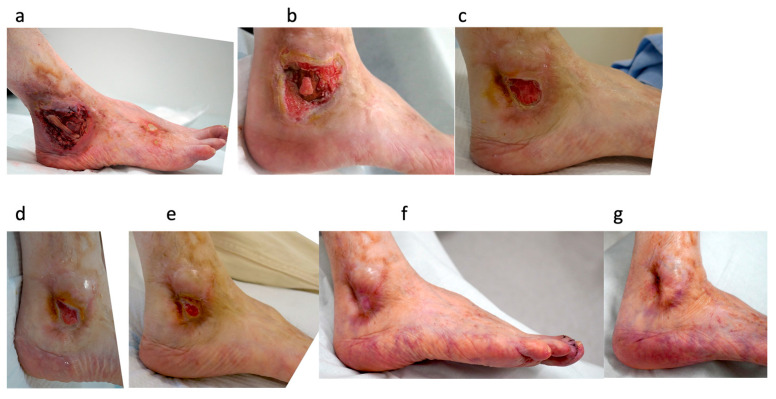
Case 8: A 65-year-old woman with a vasculitic ulcer on the right lateral malleolus. (**a**) Foot status at the first visit to our department. (**b**) After three months of conventional treatment at our department. The fibula remained exposed. (**c**) After 240 days of conventional treatment, the wound had reduced in size, but the fibula remained exposed. (**d**) After the first platelet-rich plasma (PRP) treatment. (**e**) After the sixth PRP treatment. (**f**) Seventy-four days after the first PRP treatment, the wound was completely healed. (**g**) Six months after healing, no recurrence was observed.

The patient had polyarteritis nodosa and had been on long-term steroids (prednisolone 7.5 mg). Before referral to our department, she had received wound care in another department of the university for 30 days. Upon presentation, conventional treatment was performed, including bFGF application, infection control, revascularization via EVT, debridement, and wound bed preparation using NPWT. Despite 204 days of conventional treatment, the fibula remained exposed, and the patient continued to experience severe pain. PRP therapy was then initiated. After the first PRP treatment, epithelialization at the wound edge was observed, accompanied by a dramatic reduction in pain. PRP was administered once weekly for 6 weeks. The wound healed completely 74 days after the first PRP treatment, with no recurrence observed 6 months after healing. See Figure 3.

#### 3.3.2. Case 11: 56-Year-Old Man, Vasculitic Ulcer on the Left First Toe (Figure 4)

**Figure 4:**
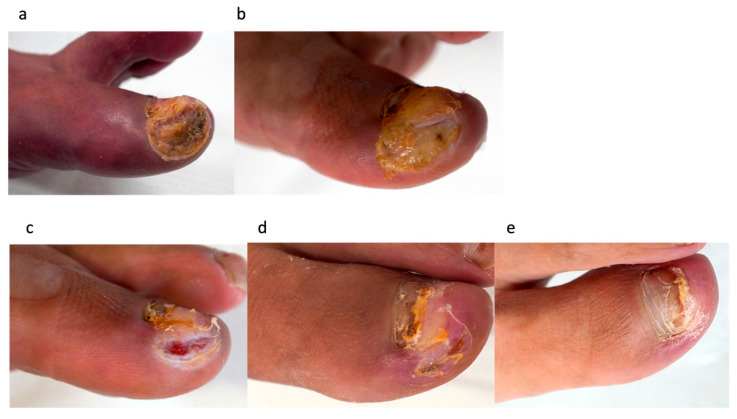
Case 11: A 56-year-old man with a vasculitic ulcer on the left first toe. (**a**) Foot status at the first visit to our department. (**b**) Sixty-two days after conventional treatment at our department. (**c**) One week after the second PRP treatment, just before the third session. (**d**) The wound was completely healed 28 days after the first PRP treatment. (**e**) Six months after healing, no recurrence was observed.

The patient had IgA nephropathy and had been on long-term use of steroids (5 mg). Before his first visit to our department, he had received treatment at a different clinic for 30 days. His toes were cold; however, revascularization was not indicated, as the ischemia was due to microcirculatory dysfunction caused by vasculitis. Conventional treatment, including maintenance debridement combined with bFGF therapy, was administered for 62 days in the outpatient clinic, but the wound did not improve. Complete wound healing was achieved after the third PRP treatment, with a total wound healing time of 28 days. No recurrence was observed 6 months after healing. See Figure 4.

#### 3.3.3. Case 5: 83-Year-Old Male, CLTI of the Right First Toe (Figure 5)

**Figure 5:**
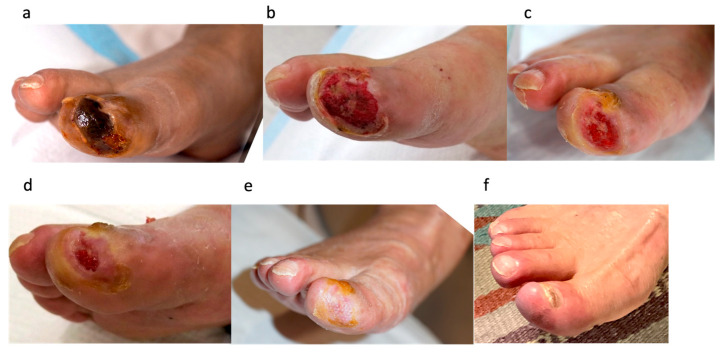
Case 5: An 83-year-old man with chronic limb-threatening ischemia of the right first toe. (**a**) Foot status at the first visit to our department. (**b**) After 101 days of conventional treatment, debridement was performed; however, no blood flow was observed. (**c**) After the second PRP treatment. (**d**) After the third PRP treatment. (**e**) The wound healed after the fifth PRP treatment. (**f**) Six months after healing, no recurrence was observed.

The patient had diabetes and was undergoing dialysis. After receiving treatment at another clinic for 14 days, he was referred to our department. At the first visit, the Wound, Ischemia, and foot Infection (WifI) classification was W2, fI0, I2. Although the patient was ischemic, revascularization was not indicated because the ischemia was due to microvascular disease. After 101 days of conventional treatment at our department, including low-density lipoprotein/fibrinogen apheresis (Rheocarna^®^) [11], debridement was performed; however, no blood flow was observed, so PRP treatment was initiated. Following PRP therapy, the wound improved and rapidly decreased in size. Complete healing was achieved after the fifth PRP treatment, with a total wound healing time of 51 days. No recurrence was observed 6 months after healing. See Figure 5.

## 4. Discussion

The concept of tissue, infection/inflammation, moisture balance, and edge of the wound (TIME) was introduced in 2003 to provide a structured framework for wound bed preparation and to optimize chronic wound management [3]. It was later updated to reflect advances in knowledge and technology, such as the recognition of biofilms, the use of NPWT, the evolution of topical antiseptic therapy for dressings and wound lavage, and expanded insights into the molecular biological processes underlying chronic wounds [12]. Since its introduction, wound care based on the TIME concept has become widely adopted globally. However, despite the relative standardization of chronic wound management, some patients continue to experience prolonged non-healing wounds. DFUs classified as stage 4 according to the WIfI system may take an average of 190 days to heal [13]. VLUs managed with appropriate compression therapy for 12 weeks show healing rates of 32–55%, increasing to up to 68% after 24 weeks. Nevertheless, 12–47% of VLUs treated for over 12 months may still fail to heal [2].

In 2019, the TIME was updated to TIMERS by integrating two additional components: Repair and Regeneration (R) and Social Factors (S) [2]. This guideline recommends that any wound failing to reduce in size by 40–50% after 4 weeks of good SWC should be classified as a hard-to-heal wound, referred to a wound care specialist or multidisciplinary team (MDT), and considered for advanced therapies. This recommendation is based on the evidence that a DFU remaining open for 30 days is associated with a 4.7-fold increase in the risk of infection, highlighting the importance of early intervention [14]. Advanced therapies, selected according to the expertise available within the complex wound clinic/MDT, should be implemented based on the outcomes of patient and wound assessment. The European Wound Management Association defines advanced therapies for wound management as those based on novel principles or technologies with demonstrated efficacy supported by comparative evidence [15].

PRP therapy is one of the advanced treatments in the ‘regeneration’ category [16,17]. Wound healing is a process in which damaged tissue is regenerated or repaired through four distinct but often overlapping stages: hemostasis, inflammation, proliferation, and maturation. Wounds become chronic when the normal healing process is disrupted. Chronic leg ulcers were defined as those that show no tendency to heal after 3 months of appropriate treatment or fail to heal completely within 12 months. During the healing process, chronic wounds exhibit a prolonged inflammatory response because growth factors and cytokines become imbalanced and dysregulated. This imbalance cannot be reversed by conventional standard wound treatments. Platelets play crucial roles in hemostasis, tissue regeneration, and host defense.

Numerous proteins are contained in the alpha granules of platelets, including PDGF, TGF-β, VEGF, epidermal growth factor, insulin-like growth factor, fibronectin, factor V, and factor VIII (von Willebrand factor). In contrast, certain bioactive molecules such as thromboxane A2 are synthesized de novo upon platelet activation, and calcium is primarily stored in dense (delta) granules rather than alpha granules.

**Figure 6:**
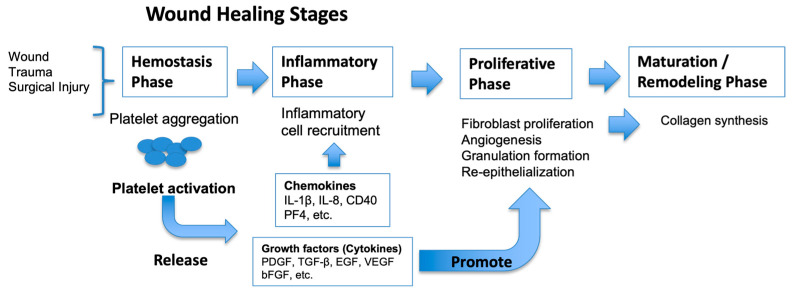
Four phases of wound healing and the mechanisms of platelet. (This figure was modified from Kusumoto K., Nankodo; 2017 [18].).

Based on these components, PRP supplements the wound with essential factors, helping to restore the balance of the wound microenvironment and support progression through the normal wound healing process (Figure 6).

In addition to growth factors, platelets secrete specific chemotactic signals that attract leukocytes to the wound site, where they exert antimicrobial activity and contribute to wound debridement [16]. Platelets also play a role in infection control, as they have a direct bacteriostatic effect on certain bacterial strains [17]. Coagulation can be induced with PRP to form a ‘white’ clot that can be placed in the wound bed. This clot serves as a primary scaffold to which cells are attracted (migration), multiply (proliferation), and release numerous growth factors. By applying PRP directly to the wound site, a moist wound environment is maintained, a biological matrix that supports new tissue growth is provided, and platelet-derived growth factors and other bioactive substances are brought into contact with the wound bed, thereby promoting the natural healing process.

In the United States, the Food and Drug Administration has approved the marketing of several PRP separation kits, including the Aurix System, which can be used to prepare PRP gel [4,5,6,7] (Table 4). PRP prepared with the Aurix achieves a near-physiological platelet concentration (1.3 × baseline). In the United States, Aurix is indicated for use in exuding wounds, such as leg ulcers, PUs, and DFUs. Its efficacy and safety were confirmed in a double-blind randomized controlled trial in patients with DFUs [4]. Based on this background, an open-label, single-arm, multicenter clinical trial was conducted at 15 centers in Japan to evaluate the efficacy and safety of PRP gel in patients with DFU [19]. The PRP gel was applied twice weekly for 8 weeks. After the removal of the PRP gel, SWC, excluding bFGF, was provided until the next application. Among the 47 patients with DFUs that failed to respond to at least 4 weeks of SWC (defined as wound area reduction rate < 50%), high rates of wound area (72.8%) and volume (92.7%) reduction were observed at the final evaluation of week 8 (day 57). The median times to possible wound closure by secondary intention and using a relatively simple procedure were 57 and 43 days, respectively. Complete wound closure was achieved in 27 patients (57.4%) at the final evaluation. No safety concerns were reported. As a result of this study, PRP gel treatment received regulatory approval and has been covered by Japan’s National Health Insurance since October 2024. In clinical practice, it is marketed in Japan as the AutoloGel System^®^. Compared with the United States Aurix System, the AutoloGel System uses a reduced concentration of calcium chloride 2% vs. 10%) and ascorbic acid (250 mg/mL to 500 mg/mL). PRP treatment is covered by insurance for up to two courses per patient (one course = 4 weeks).

Despite the small sample size, our data showed a favorable wound healing rate compared with previous results using the Aurix System [4,5,6,7]. The efficacy of PRP for DFU, VLU, PUs, and surgical wound dehiscence has been reported in numerous studies; however, investigations of vasculitic wounds remain limited [20]. Vasculitic ulcers remain among the most challenging chronic wounds. Cutaneous vasculitis encompasses several inflammatory disorders that affect microvessels within the skin, including the arterioles, capillaries, and postcapillary venules. Damage to these microvessels can impair blood flow, cause focal ischemia, and lead to the formation of skin ulcers. The prevalence of leg ulcers ranges from 0.18 to 1.0% [21]. A recent study suggested that PRP contains VEFG signaling proteins that upregulate VEGFR2 expression in vitro [22]. PRP contains multiple angiogenic growth factors, including VEGF, PDGF, and FGF, which are involved in endothelial cell signaling pathways. Previous studies have suggested that PRP may influence VEGF–VEGFR2 signaling and downstream pathways such as PI3K/Akt, p38 MAPK, and ERK1/2, which are associated with endothelial cell proliferation, migration, and angiogenesis [22]. In inflammatory vascular conditions such as vasculitis, endothelial injury and persistent inflammatory signaling may impair VEGFR2 expression and downstream signaling, resulting in reduced angiogenic responses and tissue ischemia. In patients with ischemic foot, macrovascular ischemia can often be improved by EVT. However, even after technically successful revascularization, microcirculatory dysfunction may persist due to impaired angiogenesis or inflammatory microvascular damage. This phenomenon, often described as persistent microvascular ischemia despite restored arterial inflow, represents a major barrier to tissue recovery. In this context, PRP may help restore impaired VEGF signaling by enhancing VEGFR2 activity and reactivating its downstream pathways, thereby promoting neovascularization and improving microcirculation. In addition, PRP has been reported to exert anti-inflammatory effects by modulating inflammatory cytokines and suppressing M1 macrophage polarization while promoting M2 polarization [23]. Importantly, PRP may therefore bridge the gap between macrovascular revascularization and microvascular regeneration in chronic ischemic wounds. PRP treatment using an autologous system can be completed in approximately 15 min, including blood collection, centrifugation, PRP gel preparation, and application, highlighting its practicality in clinical settings. In some cases, clinical improvement was observed after a single treatment, suggesting a potential therapeutic effect. These findings suggest that PRP gel therapy may represent a promising adjunctive option for refractory wounds, including those associated with vasculitis. However, these findings should be interpreted as exploratory, and further prospective controlled studies are required to establish efficacy.

This study had several limitations. First, its retrospective single-center design and small sample size limit the generalizability of the findings. The cohort included patients with heterogeneous etiologies, such as DFUs, vasculitic ulcers, CLTI, VLUs, and PUs, which may exhibit distinct healing dynamics. Although consistent improvement across etiologies is encouraging, disease-specific subgroup analyses were not performed. In addition, some patients in this cohort were receiving dialysis, which may affect wound healing through impaired microcirculation and systemic inflammation. Although dialysis is common among patients with CLTI in Japan and reflects real-world practice, its potential impact on treatment outcomes should be considered when interpreting the results. Additionally, variations in concomitant treatments before and during PRP therapy may have influenced the observed outcomes, making it difficult to isolate the specific effect of PRP.

Second, the comparison between healing during conventional therapy and after PRP initiation should be interpreted with caution, as these represent sequential phases of the same wound rather than independent groups. This within-subject comparison may be influenced by temporal factors, including the natural course of wound healing, regression to the mean, and the cumulative effects of prior treatments. Third, the comparison between conventional therapy and PRP was not randomized, without a proper control group; thus, potential selection bias and confounding factors cannot be excluded. Many patients had already received prolonged treatment at other institutions, with wide variations in the duration and quality of prior care. In addition, the comparison between conventional therapy duration and post-PRP healing time represents different phases of wound evolution and is therefore methodologically limited and prone to bias. Fourth, this study lacked a standardized quantitative assessment of wound size reduction, perfusion status, and microcirculatory parameters, which could have provided deeper insights into mechanisms of PRP, particularly in vasculitic or microangiopathic ulcers. Fifth, treatment frequency was constrained by the current insurance system in Japan, because frequent administration of PRP therapy is not sufficiently reimbursed. Therefore, the efficacy of alternative dosing regimens could not be evaluated. Finally, wound healing was defined as complete epithelialization, assessed by the corresponding author, which may introduce observer bias. The absence of blinded or independent outcome assessment may have influenced the evaluation of healing outcomes.

## 5. Conclusions

In this retrospective study, weekly autologous PRP gel therapy prepared using the AutoloGel System^®^ was associated with improved healing outcomes in refractory chronic wounds in an Asian population. Favorable outcomes were observed in traditionally difficult-to-treat conditions such as vasculitis-associated and microangiopathic ischemic ulcers. These findings suggest a potential role of PRP in promoting angiogenesis and improving microcirculatory perfusion in wounds associated with microvascular dysfunction. PRP may therefore represent a promising adjunctive therapy in this clinical context. However, these findings should be interpreted as exploratory, and further prospective controlled studies are required to establish efficacy.

## Figures and Tables

**Table 1:** Baseline characteristics of patients (*n* = 20).

Case	Diagnosis	Age	Sex	Diabetes	Dialysis	Wound Localization
1	DFU	59	M	+	−	Foot
2	DFU	62	M	+	−	Foot
3	DFU	38	M	+	−	Foot
4	DFU, vasculitic ulcer (RA)	70	F	+	−	Foot
5	CLTI W2fI0I2	83	M	+	+	Foot
6	CLTI W2fI0I2	82	M	+	−	Foot
7	CLTI W3fI0I2	67	M	+	+	Lower leg
8	Vasculitic ulcer (polyarteritis nodosa)	65	F	−	−	Foot
9	Vasculitic ulcer (systemic lupus erythematosus)	57	M	−	−	Lower leg
10	Vasculitic ulcer (schleroderma)	67	M	+	−	Foot
11	Vasculitic ulcer (IgA nephropathy)	56	M	−	−	Foot
12	Vasculitic ulcer (ulcerative colitis)	69	M	−	−	Foot
13	VLU	48	F	−	−	Lower leg
14	VLU	32	M	+	−	Lower leg
15	VLU	54	F	−	−	Lower leg
16	SSI	74	M	−	−	DFU Lower leg
17	SSI	76	F	−	−	Back
18	SSI	69	M	−	−	Chest
19	SSI	43	M	−	−	Lower leg
20	PU	31	M	−	−	Ischium

DFU: diabetic foot ulcer; RA: rheumatoid arthritis; CLTI: chronic limb-threatening ischemia; VLU: venous leg ulcer; SSI: surgical site infection; PU: pressure ulcer.

**Table 2:** Clinical outcomes of PRP therapy (*n* = 20).

Case	(A)	(B)	(C)	No. of PRP Application
1	21	45	81	4
2	3396	38	67	6
3	5	40	17	1
4	719	80	118	6
5	14	101	51	5
6	14	181	28	3
7	-	71	21	2
8	30	204	74	6
9	-	38	14	2
10	50	70	69	6
11	30	62	28	3
12	17	31	43	4
13	51	101	32	3
14	30	55	14	2
15	51	80	31	3
16	-	35	28	4
17	550	351	95	6
18	35	58	51	8
19	-	46	45	4
20	912	57	51	8

A: wound duration before referral (day); B: duration of conventional therapy by a wound specialist (day); C: healing time after PRP initiation (day); PRP: platelet-rich plasma.

**Table 3:** Comparison of healing duration between conventional and PRP treatment. (*n* = 20).

Statistic/Test		(B) Conventional Treatment	(C) PRP Treatment	*p*-Value
Wilcoxon signed-rank test	Median (IQR)	60.0 (43.8–85.2) days	44 (28.0–67.5) days	0.0107

**Table 4:** Summary of previous Aurix data compared with the present study [4,5,6,7,19].

	PRP	Application	Wound Healing Rate	Country, Study Design	No of Patients, Wounds	Wound Type
Our data	AutoloGel (Japan)	One a week	100% at 12 wks, WHT: 47.9 ± 28.6 days	Japan, single center, retrospective	20 pts, 20 wounds	mix
2006 Driver [4]	Aurix *	Twice a week	68.4% (13/19) at 12 wks	USA, Multicenter, prospective RCT	19 pts, 19 wounds	DFU
2019 Guide [5]	Aurix	Twice a week for 2 wks, then once a week, total 12 wks	PRP48.5% at 12 wks	USA, Multicenter RCT	66 pts, 66 wounds	DFU
2011 de Leon [7]	Aurix *	Once or twice per week	90.5% reduced of 63.6% in volume at 2.2 wks, 2.8 PRP treatment	USA, Multicenter, Observational case series	200 pts, 285 wounds	mix
2011 Sakata [6]	Aurix *	Once a week (Occasionally, twice a week)	83% at 145 days (*p* = 0.00002)	Japan, Multicenter retrospective study	39 pts 40 wounds	DFU 34 CLTI 5 PU1
2024 Ohura [19]	AutoloGel (Japan)	Twice a week	8 weeks 27/38 (57.4%)	Japan, Multicenter clinical study	47 pts, 47 wounds	DFU

*: In the original paper, it is described as “AutoloGel (Cytomedix, Inc.),” the former name of Aurix in the US.

## Data Availability

All data analyzed in this study are available from the corresponding author upon request.

## Abbreviations

The following abbreviations are used in this manuscript:

bFGF	Basic Fibroblast Growth Factor
CLTI	Chronic Limb-Threatening Ischemia
DFU	Diabetic Foot Ulcer
EVT	Endovascular Therapy
MDT	Multidisciplinary Team
NPWT	Negative Pressure Wound Therapy
PAD	Peripheral Arterial Disease
PDGF	Platelet-Derived Growth Factor
PU	Pressure Ulcer
PRP	Platelet-Rich Plasma
SWC	Standard Wound Care
TGF-β	Transforming Growth Factor-β
VEGF	Vascular Endothelial Growth Factor
VLU	Venous Leg Ulcer
WIfI	Wound, Ischemia, and foot Infection classification system
